# Prognostic Matrisomal Gene Panel and Its Association with Immune Cell Infiltration in Head and Neck Carcinomas

**DOI:** 10.3390/cancers13225761

**Published:** 2021-11-17

**Authors:** Yuri Belotti, Su Bin Lim, Narayanan Gopalakrishna Iyer, Wan-Teck Lim, Chwee Teck Lim

**Affiliations:** 1 Institute for Health Innovation and Technology, National University of Singapore, 14 Medical Drive, Singapore 117599, Singapore; yuri_belotti@bii.a-star.edu.sg; 2 Department of Biochemistry and Molecular Biology, Ajou University School of Medicine, Suwon 16499, Korea; sblim@ajou.ac.kr; 3 Department of Head and Neck Surgery, National Cancer Centre Singapore, 11 Hospital Drive, Singapore 169610, Singapore; gopaliyer@singhealth.com.sg; 4 Duke-NUS Medical School, 8 College Road, Singapore 169857, Singapore; 5 Division of Medical Oncology, National Cancer Centre Singapore, 11 Hospital Drive, Singapore 169610, Singapore; 6 Institute of Molecular and Cell Biology (IMCB), Agency for Science, Technology and Research (A*Star), 61 Biopolis Drive, Proteos, Singapore 138673, Singapore; 7 Department of Biomedical Engineering, National University of Singapore, 4 Engineering Drive 3, Singapore 117583, Singapore; 8 Mechanobiology Institute, National University of Singapore, 5A Engineering Drive 1, Singapore 117411, Singapore

**Keywords:** extracellular matrix, head and neck cancer, bioinformatics, TCGA, HPV, prognostic biomarker

## Abstract

**Simple Summary:**

Squamous cell carcinoma of the head and neck (SCCHN) is a heterogeneous group of tumors arising from squamous cells lining different anatomic sites. This type of malignancy has been mainly investigated by focusing primarily on tumor cells, but recent evidence highlighted the importance of the tumor microenvironment (TME) in cancer growth, progression and metastasis. Hence, we hypothesized that dysregulated matrisomal components could have a common association with patient survival, irrespective of the subsite of origin of the SCCHN. Using bioinformatic methods and public datasets, we successfully identified a gene panel with prognostic value in HPV-negative and non-metastatic node-negative tumors and demonstrated its association with immune cell infiltration.

**Abstract:**

Squamous cell carcinoma of the head and neck (SCCHN) is common worldwide and related to several risk factors including smoking, alcohol consumption, poor dentition and human papillomavirus (HPV) infection. Different etiological factors may influence the tumor microenvironment and play a role in dictating response to therapeutics. Here, we sought to investigate whether an early-stage SCCHN-specific prognostic matrisome-derived gene signature could be identified for HPV-negative SCCHN patients (*n* = 168), by applying a bioinformatics pipeline to the publicly available SCCHN-TCGA dataset. We identified six matrisome-derived genes with high association with prognostic outcomes in SCCHN. A six-gene risk score, the SCCHN TMI (SCCHN-tumor matrisome index: composed of MASP1, EGFL6, SFRP5, SPP1, MMP8 and P4HA1) was constructed and used to stratify patients into risk groups. Using machine learning-based deconvolution methods, we found that the risk groups were characterized by a differing abundance of infiltrating immune cells. This work highlights the key role of immune infiltration cells in the overall survival of patients affected by HPV-negative SCCHN. The identified SCCHN TMI represents a genomic tool that could potentially aid patient stratification and selection for therapy in these patients.

## 1. Introduction

SCCHN comprises a heterogeneous group of tumors arising from squamous cells lining different anatomic sites within the upper aerodigestive tract such as the nasal cavity, paranasal sinuses, lips, oral cavity, oropharynx, hypopharynx or larynx [1,2]. The global incidence rates have increased by 36.5% between 2005 and 2015 [3]. SCCHN predominantly affects people above 50 years old, with incidence rates higher among men than women [4]. Tobacco and alcohol exposure constitutes the major risk factors for the development of such cancers [5,6]. Human papillomavirus (HPV) is an important etiologic factor of SCCHN [7]. Aside from etiology, both tumor staging and pathological features have prognostic value [8]. The presence of metastases and aggressive pathological features such as extranodal extension (ENE), perineural invasion (PNI) or lymphovascular invasion (LVI) are prognostic factors for reduced survival [9,10,11]. Subsite specific etiological factors and associated tumor and microenvironment differences may influence the clinical outcomes of existing modalities of treatment [12,13]. Recent advancements in high throughput molecular profiling have also added new prognostic markers [14,15] that relate to the biology of the specific anatomical subsite of interest, as a consequence of their specific underlying molecular pathways [16]. This could account for the inherent heterogeneity of this malignancy and aid prognostication and possibly treatment selection and intervention.

Epithelial malignancies have been extensively investigated at the genomic and epigenomic levels focusing primarily on tumor cells. Recent evidence increasingly highlights the importance of the tumor microenvironment (TME) in cancer growth, progression and metastasis [17,18]. Therefore, a deeper understanding of the role of the cancer-associated extracellular matrix (ECM) components might help to identify new diagnostic and prognostic tools. In 2015, Naba, A. et al. [19] identified a list of 1068 human ECM genes encoding ECM and ECM-associated proteins and presented omics data indicating their roles in development, homeostasis and disease.

Bioinformatic approaches are powerful tools that enable whole-genome investigation of the abnormalities exhibited by cancer tissues from large groups of patients. Hence, in this study, using a series of recently developed web-based tools and open-source software, a bioinformatic-based study on a transcriptomic dataset publicly available in the “The Cancer Genome Atlas (TCGA)” [20] database was conducted. First, based on recent studies [21,22], we hypothesized that dysregulated matrisomal components could have a common association with patient survival, irrespective of the subsite of origin of the SCCHN. Specifically, transcripts that were associated with survival in HPV-negative and non-metastatic node-negative tumors, were examined to minimize confounding by treatment, stage, and etiology. Next, we defined a novel prognostic signature, the SCCHN-tumor matrisome index (SCCHN TMI), assessed its prognostic ability across independent datasets and its association with immune cell infiltration.

## 2. Materials and Methods

### 2.1. SCCHN TCGA Data

A list of 1068 human matrisome genes, first published by Naba, A. et al. [19] and more recently revised [23], was retrieved from the M.I.T. “Matrisome Project” website (www.matrisomeproject.mit.edu/other-resources/human-matrisome, accessed on 10 March 2021) and loaded into the web-based tool XENA [24] (www.xenabrowser.net, accessed on 10 March 2021). The study “TCGA Head and Neck Cancer (HNSC) study” was selected: (1) as “first variable” the “Genomic” data type was selected; (2) the matrisomal genes inputted and (3) the normalized gene expression selected. (4) Relevant “phenotypic” data were selected to investigate the various clinical covariates.

### 2.2. SCCHN scRNA-Seq Data

Transcript-level expression values (TPMs) for 23,686 genes, across 5902 cells derived from 18 SCCHN patients, including five matched pairs of primary tumors and lymph node metastases [25], were re-analyzed in this study to identify specific cell types expressing SCCHN TMI signatures. Scaling of the data and linear dimensional reduction were performed using R (v 4.0.3) “Seurat” package (v 4.0.1). Cells that were annotated with the identified cell type by the authors of the original work (i.e., cells that are classified as “cancer cell”, “B cell”, “dendritic”, “endothelial”, “fibroblast”, “macrophage”, “mast”, “myocyte”, or “T cell”) were included in the analysis.

### 2.3. Construction of the SCCHN TMI Risk Score

Expression levels and clinical annotation for the HPN^−^, N0 subgroup were selected and imported into R (v4.0.2) and RStudio (v1.3.1073) where the package “RegParallel” (v1.8.0) was used to fit the Cox proportional hazard model independently for each gene. The genes characterized by log-rank *p*-value < 0.01 were selected to generate the SCCHN-specific prognostic signature (the SCCHN-tumor matrisome index), combining the expression level and the Cox regression coefficient (Betai) associated with the prognostic genes: HNSCC TMI = $\sum_{i}\mathrm{Expression}({\mathrm{Gene}}_{i})\cdot {\mathrm{Beta}}_{i}$. The effect on overall survival probabilities of the HNSCC TMI scores was assessed using a Cox proportional hazard model (using the “coxph” function of the “survival” package (v3.2-11) in R/Bioconductor [26]). The Cox proportional hazard assumption was checked by the scaled Schoenfeld residual test using the cox.zph function provided by the survival package in R/Bioconductor [26].

### 2.4. Patient Stratification and Survival Analysis

A median cut-off was used to stratify patients into low- and high-risk groups. Kaplan−Meier (KM) survival curves were generated to test the prognostic value of the SCCHN TMI. Overall survival (OS) time was computed from the date of surgery until death. The survival analysis was conducted using the “survival” package. The log-rank *p*-value was indicated in each KM curve and considered statistically significant if smaller than 0.05.

### 2.5. Validation

The prognostic value of the SCCHN TMI was internally and externally validated using the penalized Cox model and represented via time-dependent AUCs using the R packages “hdnom“ (v6.0.0) and “rms” (v6.2-0). The internal validation was performed using elastic-net with “Bootstrap validation” penalty trade-off parameter α = 0.05, regularization parameter λ = 1.763886. The external validation was conducted on the SCCHN GEO dataset (accession number GSE65858).

This dataset was generated by Wichmann et al. [27] and contains gene expression data from 270 patients diagnosed with SCCHN collected using the platform GPL10558 Illumina HumanHT-12 V4.0 expression Beadchip. The dataset was accessed using Phantasus (v.1.9.2, https://artyomovlab.wustl.edu/phantasus/, accessed on 11 March 2021) where “Log2” and “quantile normalization” adjustments were applied to the data. Moreover, to remove lowly-expressed probes and ensure only one row per gene in the gene expression matrix, data were collapsed using “Maximum Median Probe” with “gene symbol” as the collapse field. The dataset was then downloaded as a spreadsheet file for further analysis.

### 2.6. Role of the Proteins Encoded by the SCCHN TMI Genes

The role of the proteins encoded by the SCCHN TMI genes were assessed using the Human Protein Atlas web tool (www.proteinatlas.org, accessed on 15 March 2021).

### 2.7. Network Analysis

The web-based tool NetworkAnalyst (https://www.networkanalyst.ca, accessed on 12 March 2021) was used to perform the network analysis of the 6 HNSC-TMI genes. Specifically, the “Gene Regulatory Network, Signaling Network” option was selected. Data from the SIGnaling Network Open Resource (downloaded on 12 March 2021) were analyzed.

### 2.8. Tumor-Infiltrating Immune Cell Analysis

The web-based tool CIBERSORTx (https://cibersortx.stanford.edu/, accessed on 6 May 2021) was used to estimate the relative fraction of 22 immune cell types based on the RNA-seq data, as a function of the SCCHN TMI risk group. We selected LM22 (22 immune cell types) for the signature gene file, 100 for permutations, and disabled quantile normalization for all runs. Subsequently, box plots were generated to present the differences in infiltrated immune cells between high and low risk groups using the “ggplot2” package (v3.3.3). Two-sided, unpaired two-samples Wilcoxon test was performed between the two risk groups.

### 2.9. Machine Learning for Personalized Prediction of the Risk Group

The computational classification was performed in Orange (Version 3.29.3) using the method recently developed by Belotti et al. [28]. The SCCHN TMI risk groups were classified based on the gene expression levels of the SCCHN TMI genes. Specifically, only 5 genes were included in the analysis as one of the genes (SFRP5) is missing in the GSE65858 dataset. The algorithm workflow is shown in Figure S1. First, expression values of the SCCHN TMI genes for each patient were imported into Orange, together with the SCCHN TMI risk group previously calculated using the median cut-off. Then the data are sent to the “test and score” widget where multiple models are tested: (a) k-nearest neighbors (kNN, number of neighbors = 5, metric = Euclidean, weight = uniform). (b) Logistic regression (regularization type = lasso, strength = C1). (c) Random forest (number of trees = 10, depth of the individual tree is limited to 5, subsets smaller than 5 are not split). (d) Support vector machine (SVM, cost = 1, regression loss = 0.1, kernel = linear, numerical tolerance = 0.001, iteration limit = 100). (e) Neural network (neurons in hidden layers = 200, activation = ReLu, solver = Adam with regularization = 0.0001, maximum number of iterations = 200). The validation of the models was carried out using 10-fold cross-validation. Moreover, the classification was also validated using an independent GEO dataset (GSE65858).

## 3. Results

### 3.1. Identification of SCCHN-Specific Tumor Matrisome Index

**Figure 1:**
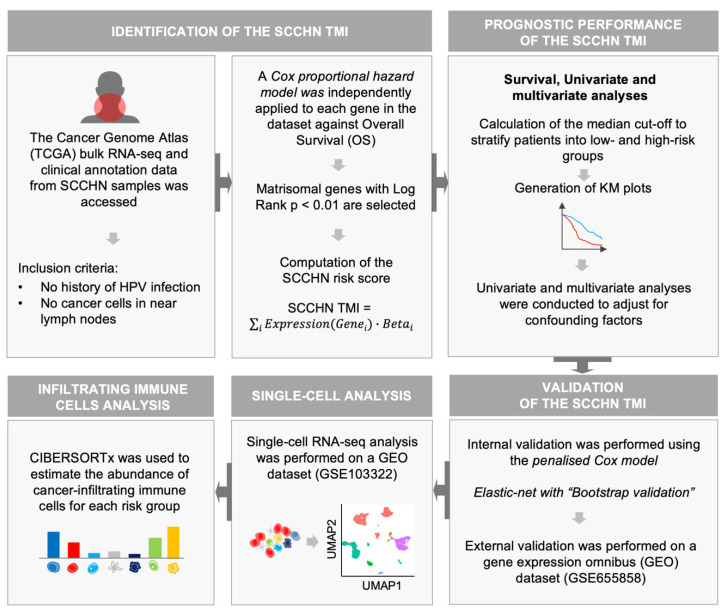
Schematic illustration of the bioinformatics workflow. We first constructed a matrisome-derived SCCHN-specific signature from primary tissue samples using a bioinformatic-based approach. Specifically, access and retrieval of large datasets from “The Cancer Genome Atlas database” was conducted via XENA browser. SCCHN-tumor matrisome index (SCCHN TMI) was computed using R: (1) prognostic genes were identified through the application of the Cox proportional hazard model; (2) the median cut-off was used to stratify patients into low and high-risk and generate Kaplan−Meier (KM) plots for each risk group; (3) internal validation and external validation of the SCCHN TMI were conducted on a microarray and scRNA-seq datasets. The web-based tool CIBERSORTx was used to estimate the relative fraction of cancer-infiltrating immune cells based on the RNA-seq data, as a function of the SCCHN TMI risk group.

A series of recently developed web-based bioinformatics tools and open-source software were utilized to access and analyze the HNSC-TCGA dataset. The analysis workflow is summarized in Figure 1. First, the matrisomal genes (see Methods) were input into XENA [24] and the normalized RNA-seq data were retrieved, together with the clinical annotation of the samples. The subgroup of patients with no HPV infection and no regional lymph node involvement (HPV^−^, N0) that underwent surgical treatment, was selected to reduce heterogeneity. A Cox proportional hazard model was independently applied for each matrisomal gene in the dataset against overall survival (OS). The genes with log rank *p* < 0.01 were selected and an SCCHN-specific tumor matrisome index (SCCHN TMI) was defined. The outcome of the Cox proportional hazard model to the matrisomal genes is shown in Table S1. Six genes were identified: *MASP1 EGFL6*, *SFRP5*, *SPP1*, *MMP8* and *P4HA1*. Based on the results derived from the Cox regression, a nomogram to predict survival probability at 2, 3 and 5 years after surgery for clinical use was developed (Figure S2A). Calibration curves for this nomogram are plotted in Figure S2B,C.

**Figure 2:**
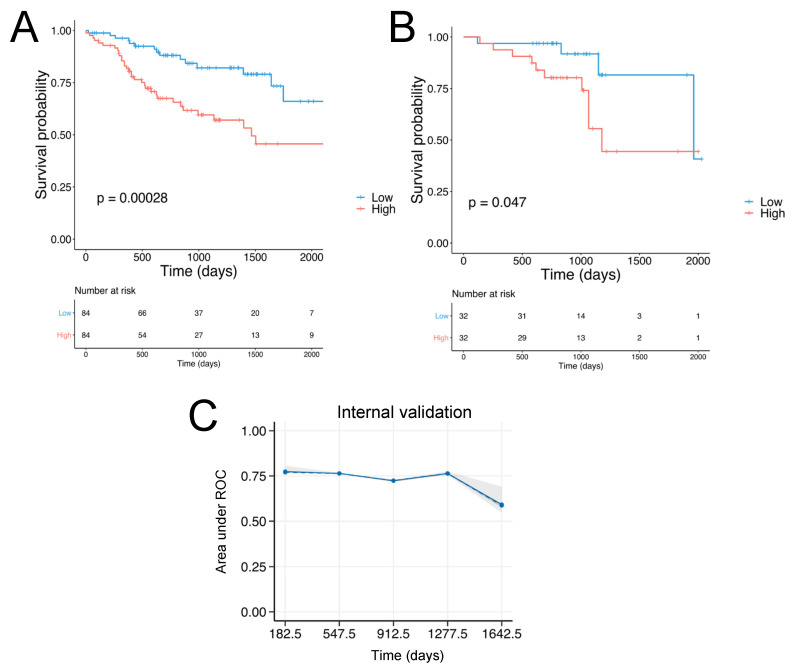
Identification of the SCHNN TMI and evaluation of its prognostic value. (**A**) Kaplan−Meier plots for the HPV^−^, N0 subgroup (*n* = 168). (**B**) Kaplan−Meier plots for the HPV^−^, N0 subgroup for the validation set (*n* = 64). (**C**) Internal validation: the performance of the elastic-net model is internally assessed by time-dependent AUC (area under the ROC curve) with “bootstrap” resampling at every year from the first 6 months to the year 4.5. The solid line represents the mean of the AUC at each time point across all bootstrap predictions, the dashed line represents the median of the AUC. The shaded interval shows the minimum and maximum of AUC.

Two of the genes composing the SCCHN TMI, *MASP1* and *EGFL6* are associated with good prognosis (HR < 1) while the other four: *SFRP5*, *SPP1*, *MMP8* and *P4HA1* are associated with poor outcomes (HR > 1), as shown in Table S1. The details of these genes are shown in Table S2. Using a median cut-off, patients were stratified into low and high-risk groups and Kaplan−Meier (KM) plots were generated, as shown in Figure 2A,B. Specifically, the group characterized by low SCCHN TMI had significantly better survival outcomes.

**Figure 3:**
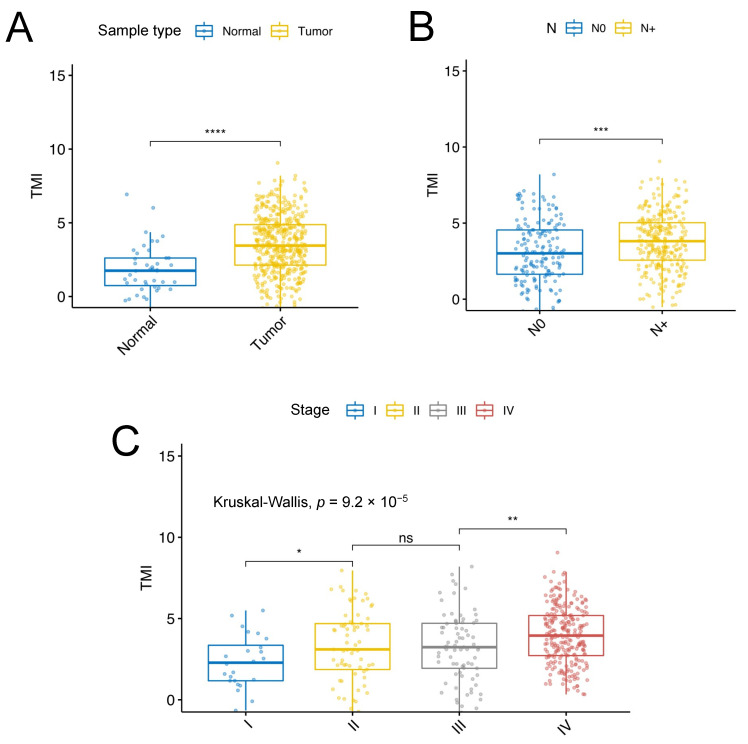
Distribution of the SCCHN TMI scores for each HPV^−^ sample as a function of clinicopathological factors. (**A**) Sample type; (**B**) absence (N0) or presence (N+) of regional lymph node invasion; (**C**) pathological stage. The whiskers indicate the range from Q1 + 1.5 × IQR to Q3 − 1.5 × IQR and the line is the median. Wilcoxon test **** *p* ≤ 0.0001, *** *p* ≤ 0.001, ** *p* ≤ 0.01, * *p* ≤ 0.05, ns: not significant.

Univariable and multivariable Cox regression analyses were performed to adjust for confounding factors such as age and gender, history of cigarette smoke and alcohol consumption, and pathological tumor (T) stage (according to AJCC version 8), as shown in Figure S3. The comparison between low and high SCCHN TMI for the HPV^−^, N0 subgroup among conventional clinical parameters is illustrated in Figure S4. Univariable survival analyses revealed that the SCCHN TMI could predict overall survival (OS), disease-specific survival (DSS), and disease-free interval (DFI), as shown in Figure S5. The individual patient’s SCCHN TMI scores exhibited statistically significant differences across different clinicopathological factors such as sample type, regional lymph node involvement and pathological stage, as shown in Figure 3. Specifically, the samples with the presence of regional lymph node invasion (N+) includes N1 (*n* = 78), N2 (*n* = 13), N2a (*n* = 11), N2b (*n* = 121), N2c (*n* = 54), N3 (*n* = 9), and NX (*n* = 89).

### 3.2. Validation of the SCCHN TMI

**Figure 4:**
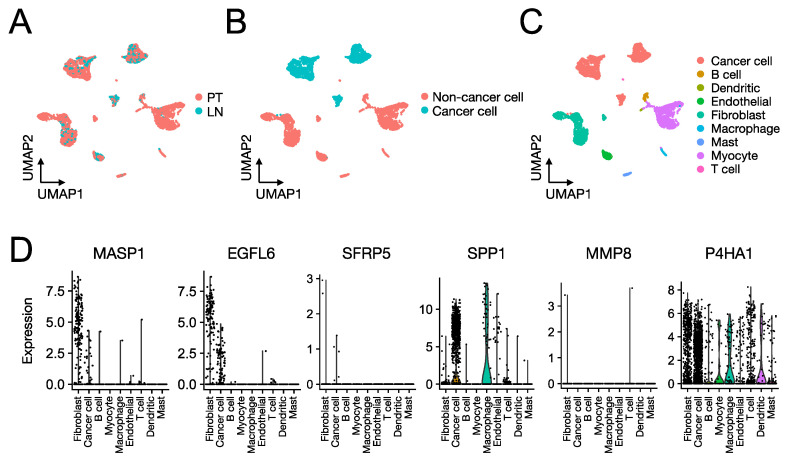
Expression of the SCCHN TMI genes at the single-cell level. UMAP (Uniform Manifold Approximation and Projection) of 5560 cells colored by (**A**) issue of origin (primary tumors (PT) and lymph node (LN) metastases), and (**B**,**C**) cell type. (**D**) Relative expression of the six SCCHN TMI genes across different cell types.

The prognostic value of the SCCHN TMI was internally and externally validated using machine-learning-based algorithms. Specifically, the internal validation was performed using the penalized Cox model. In Figure 2C and the time-dependent AUC (area under the receiver operating characteristic (ROC) curve) is shown. The Gene Expression Omnibus (GEO) dataset GSE65858 was used as a validation set. Figure 2B shows the KM plot for the validation set (*n* = 64). To identify specific cell types expressing the 6 genes composing the SCCHN TMI, we next analyzed the scRNA-seq SCCHN dataset (GSE103322), derived from 18 SCCHN patients, including matched pairs of primary tumors (PT) and lymph node (LN) metastases (Figure 4). *MASP1* and *EGFL6* were specifically expressed predominantly in subpopulations of fibroblasts and cancer cells, while *SPP1* and *P4HA1* were expressed by subgroups of several cell types composing the tumor and its matrisome, including immune cells, such as macrophages and dendritic cells, and fibroblasts. SFRP5 and MMP8 were expressed only in a small number of cells.

### 3.3. Regulatory Signaling Network Analyses

The outcome of the regulatory network analysis is shown in Figure S6A. The KEGG (Kyoto Encyclopedia of Genes and Genomes) enrichment analysis was performed for the genes identified in the signaling network (Figure S6B). Three genes (RUNX2, ERG and MMP3) are involved in “transcriptional misregulation in cancer”. ERG and MMP3 are also associated with prostate cancer. MMP3 is involved in the IL-17 signaling pathway, TNF signaling pathway, and rheumatoid arthritis. ETS2 and MMP7 are involved in HTLV-1 infection.

### 3.4. Role of the Proteins Encoded by the SCCHN TMI Genes

The gene *MASP1* encodes a serine protease that is a component of the lectin pathway, which plays an essential role in the innate and adaptive immune response [29]. This gene has been found to be a favorable prognostic marker in liver cancer [29]. *EGFL6* encodes a member of the epidermal growth factor (EGF) repeat superfamily, involved in cell cycle regulation, proliferation, and developmental processes [30]. It is a favorable marker in both ovarian and head and neck cancer [30]. *SFRP5* has a role as a modulator of Wnt signaling, which is involved in regulating cell growth and differentiation in specific cell types [31]. *SPP1* encodes a protein which is a cytokine that upregulates expression of interferon-gamma and interleukin-12, and it is an unfavorable prognostic marker in liver, pancreatic, and cervical cancer [32]. *SPP1* is involved in ECM-receptor interaction, Toll-like receptor signaling pathway, Apelin signaling pathway [32]. *MMP8* encodes a member of the Matrix metalloproteinases (MMPs), a family of proteolytic enzymes which is involved in degrading components of the extracellular matrix and promoting invasion and metastasis in various cancers. In SCCHN it has been shown that imbalances between matrix metalloproteinases and their inhibitors contribute to the progression are linked to the prognosis of the malignancy [33,34]. *P4HA1* encodes a key enzyme involved in collagen synthesis and catalyzes the formation of 4-hydroxyproline, essential for the three-dimensional folding of procollagen chains [35]. It is an unfavorable prognostic marker in renal, head and neck, cervical, pancreatic, lung and breast cancers [35].

### 3.5. Association between Tumor-Infiltrating Immune Cells and the Risk Group

**Figure 5:**
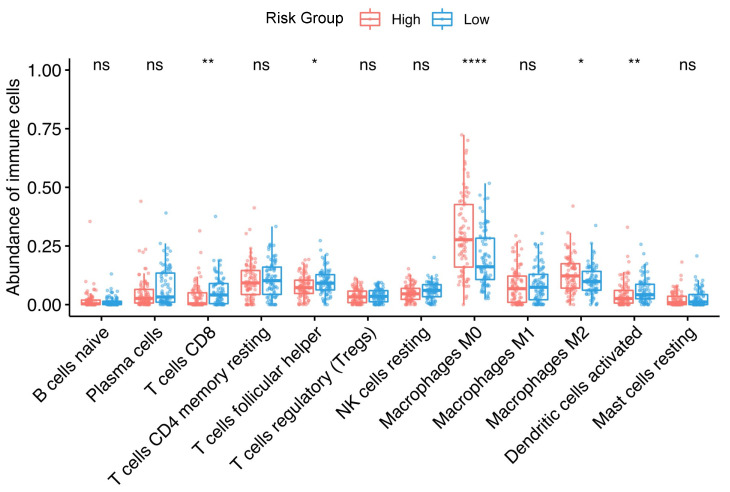
Association between SCCHN TMI risk group and infiltrative immune cells. The box plots show the percentages of infiltrative immune cells calculated by CIBERSORTx between high- and low-risk groups in the “TCGA-HNSC”, HPV^−^, N0 subgroup. The size of the boxes indicates the interquartile range IQR which spans from the first quartile (Q1) to the thirst quartile (Q3). The whiskers indicate the range from Q1 + 1.5 × IQR to Q3 − 1.5 × IQR and the line is the median. A two-sided, unpaired two-sample Wilcoxon test was performed between the two groups. **** *p* ≤ 0.0001; ** *p* ≤ 0.01; * *p* ≤ 0.05, ns: not significant.

Using CIBERSORTx the relative abundance of 22 immune cell subsets of SCCHN were estimated for the HPV^−^, N0 cohort. In Figure 5, the differences in the abundance of the infiltrative immune cells between high-risk and low-risk groups are shown. Specifically, only the immune cells with measurable abundance are shown. A statistically significant higher abundance of T cells CD8, T cells follicular helper, activated dendritic cells were found in the low-risk group, whereas a higher abundance of M0 and M2 macrophages were found in the high-risk group.

### 3.6. Machine Learning Approach for Risk Group Classification

**Figure 6:**
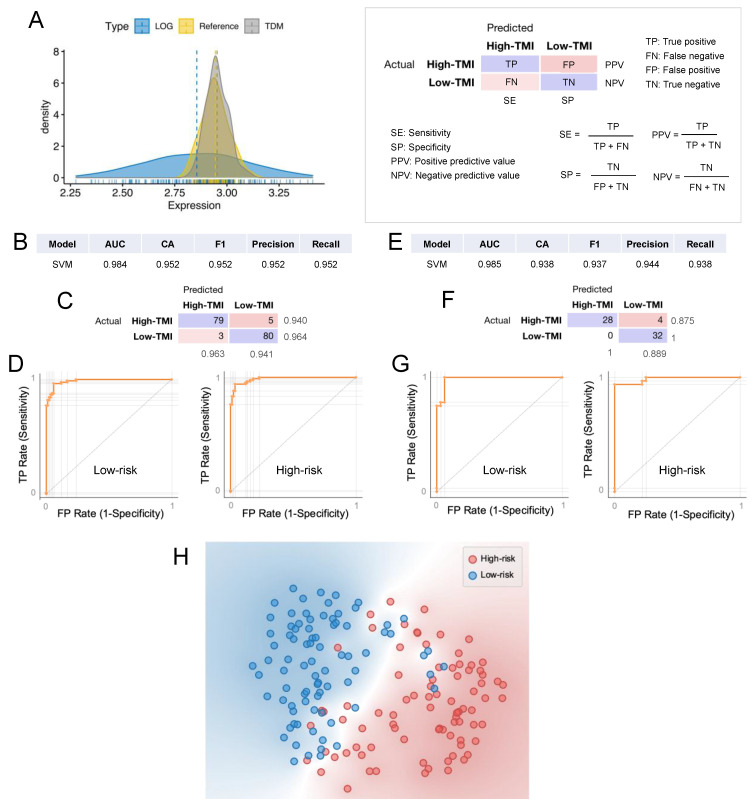
Computational classification of the SCCHN TMI risk group at the individual patient level. (**A**) Comparison between TDM and LOG-transformed training data (TCGA) in fitting the validation dataset (GSE65858) distribution. (**B**) Results of the classification model evaluation using 10-fold cross-validation. (**C**) Confusion matrix of the support vector machine (SVM) model, which best scored in the classification. (**D**) Receiver operating characteristic (ROC) curves for each of the SCCHN TMI risk groups using SVM. (**E**) Results of the classification model evaluation using the validation dataset. (**F**) Confusion matrix of the support vector machine (SVM) model. (**G**) Receiver operating characteristic (ROC) curves for each of the SCCHN TMI risk groups using SVM. AUC = area under the curve, CA = classification accuracy. (**H**) The t-SNE plot of two risk groups.

We sought to demonstrate the predictive potential of our SCCHN TMI in stratifying patients across different platforms. Given that the TCGA and GEO data are derived from RNA-seq and microarray, respectively, we used a cross-platform normalization tool to enable comparison between the two datasets of different profiling platforms. Specifically, we used TDM [36] transformation to make RNA-seq data compatible with microarray data, as recently shown [37]. Figure 6A shows the comparison between TDM and logarithmic transformation. The TDM transformation best fitted the reference microarray data (validation dataset, GSE65858) distribution. Using a supervised machine learning approach [28], we next developed SCCHN TMI-based risk group classifiers. First, we trained multiple classifiers (as shown in Figure S1) on the TDM-transformed SCCHN TCGA dataset using 10-fold cross-validation (Figure 6B–D). The best predictive model, support vector machine (SVM), was then evaluated on the validation dataset (Figure 6E–G). Both cross-validation and external validation on the GSE65858 dataset resulted in an area under the (receiver operating characteristic) curve (AUC) of 0.984 and 0.985 and classification accuracy of 95.2% and 93.8%, respectively. A clear separation between the two risk groups indicates a superior classification performance, as shown in the t-distributed stochastic neighbor embedding (t-SNE) plot (Figure 6H).

## 4. Discussion

The discovery of reliable prognostic biomarkers capable of identifying patients with a higher risk of unfavorable survival outcomes is needed in order to better define patients who might require further adjuvant treatment after surgical resection. The SCCHN TMI gene panel, which was constructed by focusing only on ECM molecules, holds potential clinical as well as biological significance. In an HPV negative subset, where the overall prognosis is poor, the SCCHN TMI was able to predict overall survival (OS), disease-specific survival (DSS), and disease-free interval (DFI). A high SCCHN TMI score was an unfavorable prognostic factor for all the analyzed endpoints. Gene ontology (GO), KEGG pathway enrichment and signaling network analyses revealed that the six SCCHN TMI genes are mostly associated with signaling networks involved in cancer-related transcriptional dysregulation and two important pathways: IL-17 and TNF. The former has already been shown to negatively correlate with the overall survival of head and neck cancer patients [38]. TNF-α, which is found in the TME, is secreted by macrophages, lymphocytes and natural killer (NK) cells and mediates the production of proinflammatory factors that elicit tumor growth and recently emerged as a promising cancer therapy target [39,40].

We found that patients in the high-risk group exhibited a proinflammatory phenotype enriched with macrophages (M0 and M2 phenotypes). Tumor-associated macrophages (TAMs) are a key component of the SCCHN tumor microenvironment as they have specific roles in regulating the immune response to cancer (refer to Evrard et al. [41] for a detailed review). Moreover, TAMs have been shown to affect cell proliferation, vascularization, stromal formation and dissolution [42]. Our results are coherent with the previous literature that highlighted the proinflammatory and tumor-promoting role of TAMs in SCCHN [43,44,45,46]. Two recent meta-analyses [43,44] found that increased densities of TAMs in the TME, particularly M2-like, correlate with poor clinicopathologic markers in SCCHN. A recent study by Tekin et al. [47] showed that M0 macrophages harbor anti-tumorigenic activities, which seem to be mediated by TNF-α which is associated with M0 macrophage-induced cell death in pancreatic cancer. Furthermore, an increasing abundance of infiltrated M0 macrophages was associated with poorer outcomes in breast cancer [48]. To our knowledge, however, our results elucidated the potential role of M0 macrophages in SCCHN for the first time.

Patients in the SCCHN TMI low-risk group exhibited an increased abundance of CD8+ and follicular helper T cells as well as activated dendritic cells. These results are consistent with prior reports which showed significantly better survival outcomes [49,50,51] in SCCHN patients with a higher abundance of infiltrative lymphocytes. Moreover, a recent work by Cillo et al. [52] found that T follicular helper cells are associated with longer progression-free survival in SCCHN patients and that the activation of dendritic cells could improve antitumor T cell responses. Hence, the SCCHN TMI might have important implications for prognosis and further adjuvant treatment decisions, as low-risk scores are associated with high levels of infiltration of antitumor T cells and low levels of infiltration of tumor-promoting TAMs. These provide potential points of therapeutic intervention that need to be validated prospectively in clinical trials of specific inhibitors.

The single-cell RNA-seq analysis of the SCCHN TMI genes revealed that four of these genes (MASP1, EGFL6, SPP1, and P4HA1) are expressed in subpopulations of fibroblasts, macrophages, T cells and in tumor cells. It is noteworthy that two genes, SPP1 and P4H41, exhibited the highest association with immune cells. Specifically, they are highly expressed in both T cells and macrophages, hence they could play an important role in linking the SCCHN risk score with the different patterns of infiltrative immune cells in the SCCHN’s TME. Two genes (SFRP5 and MMP8) were lowly expressed in this dataset. In the study by Puram et al. [25], the authors analyzed 5902 single cells from 18 patients with tumors of the oral cavity, which is one of the subsites of SCCHN. Therefore, the low expression of SFRP5 and MMP8 in this dataset might be, to some extent, attributed to the small cohort of patients included in the analysis and the presence of only one SCCHN subsite.

Using machine learning, we demonstrated high computational classification accuracy between the risk groups in the data collected using different platforms (RNA-seq and microarrays), despite the small sample size of the validation dataset. This has important clinical implications as it demonstrates the robustness of our SCCHN TMI in stratifying HPV^−^, N0 patients. Finally, statistically different expression levels were found for each SCCHN TMI gene between the two risk groups (Figure S7). As the SCCHN TMI comprises a small number of genes, their expression levels could be quantified using RT-PCR directly on postoperative specimens to conduct prospective validation studies.

One of the limitations of this study is the presence of only one validation dataset. This is due to the incomplete clinicopathological information in all the other available public datasets that we evaluated. This is a current issue in the field of SCCHN. Therefore, the assessment of the SCCHN TMI signature in multiple larger validation dataset cohorts is warranted in the future. This will enable the identification of a global cut-off value for patient stratification, and further improve the clinical utility of the SCCHN TMI. Another limitation is the use of publicly available algorithms that could change over time. This might limit the reproducibility of this study, but novel algorithms with improved quality, accuracy, usability and speed are likely to emerge in the future.

## 5. Conclusions

In conclusion, the identified SCCHN TMI gene signature represents a genomic tool that could potentially enable a better understanding of the molecular mechanisms associated with the interaction between the tumor and its microenvironment. Lastly, the SCCHN TMI could enhance patient stratification progression and selection and aid personalized intervention.

## Data Availability

The data analyzed in this study were obtained from TCGA using XENA browser, accessing the “TCGA Head and Neck Cancer (HNSC) study”. The validation dataset was obtained from GEO (Gene Expression Omnibus) under the accession codes GSE65858 and accessed using Phantasus. The scRNA-seq dataset analyzed in this study is available from GEO (Gene Expression Omnibus) under the accession code GSE103322.

## Supplementary Materials

The following are available online at https://www.mdpi.com/article/10.3390/cancers13225761/s1, Figure S1: Workflow for the machine learning prediction of the risk group, Figure S2: Nomogram and computational calibration of the model, Figure S3: Univariate and multivariate analysis, Figure S4: Comparison between low and high SCCHN TMI for the HPV^−^, N0 sub-group among conventional clinical parameters (TCGA dataset), Figure S5: Association between SCCHN TMI and clinical outcomes, Figure S6: Regulatory network analyses of the SCCHN TMI genes, Figure S7: Expression levels as a function of the SCCHN TMI risk group for each gene, Table S1: Outcome of the Cox Proportional Hazard Model to the 1068 matrisomal genes, Table S2: The 6-gene SCCHN TMI signature.
