# Supplementary material for: Prognostic Matrisomal Gene Panel and Its Association with Immune Cell Infiltration in Head and Neck Carcinomas
Source: Cancers (Basel). 2021 Nov 17;13(22):5761. doi: 10.3390/cancers13225761 (PMC8616409; doi:10.3390/cancers13225761)
Supplement: Supplementary file 1 [file cancers-13-05761-s001.zip › cancers-1418121-supplementary.pdf]

Supplementary Information

Article

Prognostic Matrisomal Gene Panel and Its Association with Immune Cell Infiltration in Head and Neck Carcinomas

Table S1. Outcome of the Cox proportional hazard model to the 1068 matrisomal genes.

Genes	Beta	Standard Error	Z	P	Log Rank	HR
**MASP1**	-0.2288919	0.06157463	-3.7173083	0.00020136	0.00013886	0.79541454
**EGFL6**	-0.3847713	0.12780185	-3.0106866	0.00260658	0.00260553	0.68060626
**SFRP5**	0.17344732	0.06223836	2.78682351	0.00532275	0.00467056	1.18939803
**SPP1**	0.15722874	0.05643049	2.78623738	0.00533238	0.00511423	1.17026326
**MMP8**	0.19925024	0.07562326	2.63477433	0.00841932	0.00789914	1.22048734
**P4HA1**	0.48488613	0.18269568	2.65406458	0.00795286	0.00792565	1.62399007

Table S2. The 6-gene SCCHN TMI signature.

Gene Symbol	Division	Category	Gene Name	Synonyms	HGNC_IDs
P4HA1	Matrisome-associated	ECM Regulators	prolyl 4-hydroxylase, alpha polypeptide I	P4HA	8546
SPP1	Core matrisome	ECM Glycoproteins	secreted phosphoprotein 1	BNSP|BSPI|ETA-1|MGC110940|OPN	11255
MASP1	Matrisome-associated	ECM Regulators	mannan-binding lectin serine peptidase 1 (C4/C2 activating component of Ra-reactive factor)	CRARF|CRARF1|DKFZp686I01199|FLJ26383|MASP|MGC12628	6901
MMP8	Matrisome-associated	ECM Regulators	matrix metallopeptidase 8 (neutrophil collagenase)	CLG1|HNC|PMNL-CL	7175
EGFL6	Matrisome-associated	Secreted Factors	EGF-like-domain, multiple 6	DKFZp564P2063|MAEG|W80	3235
SFRP5	Matrisome-associated	Secreted Factors	secreted frizzled-related protein 5	SARP3	10779

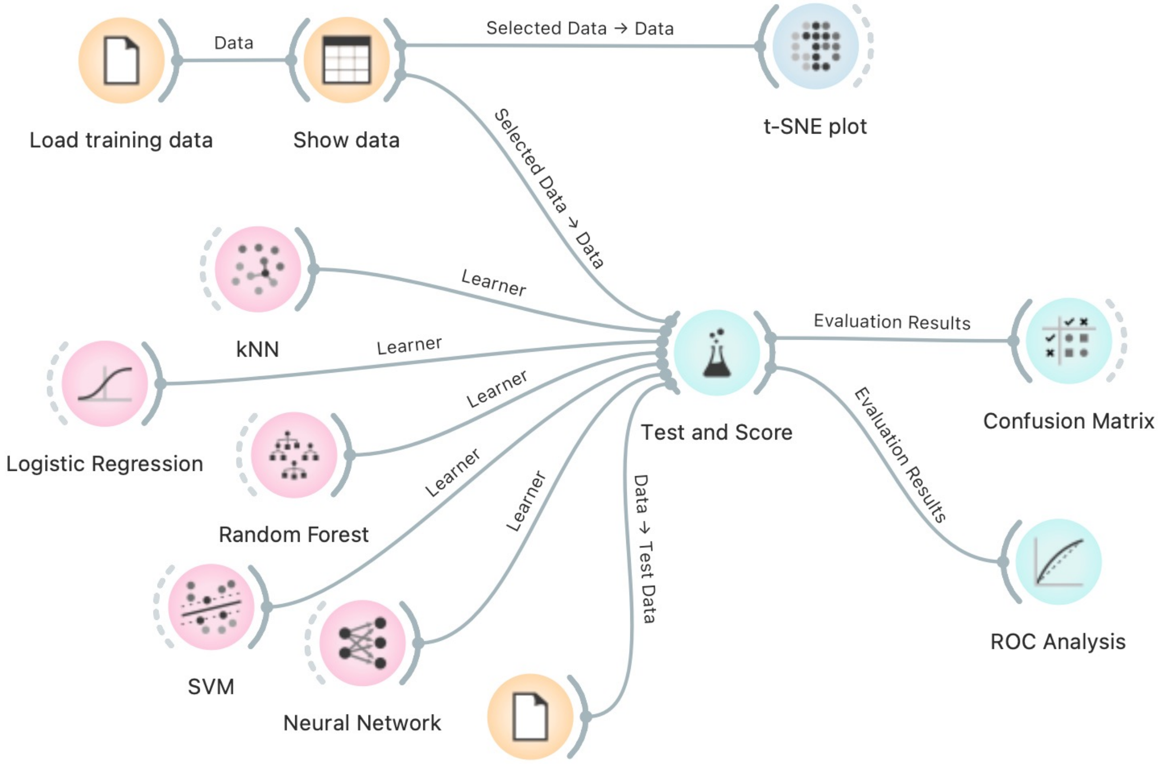

Figure S1. Workflow for the machine learning prediction of the risk group. 1) Expression values of the SCCHN TMI genes for each patient were imported into Orange together with the SCCHN TMI risk group previously calculated using the median cutoff. The TDM-transformed TCGA dataset is used as training dataset. The t-SNE plot is generated and visualised. Different machine learning models are built and trained on the data and 10-fold cross validation is performed. The predictive scores calculated for each validation for each model. Finally, the GSE65858 is loaded into Orange and used as a validation dataset. Confusion matrixes and receiver operating characteristic (ROC) curves are generated.

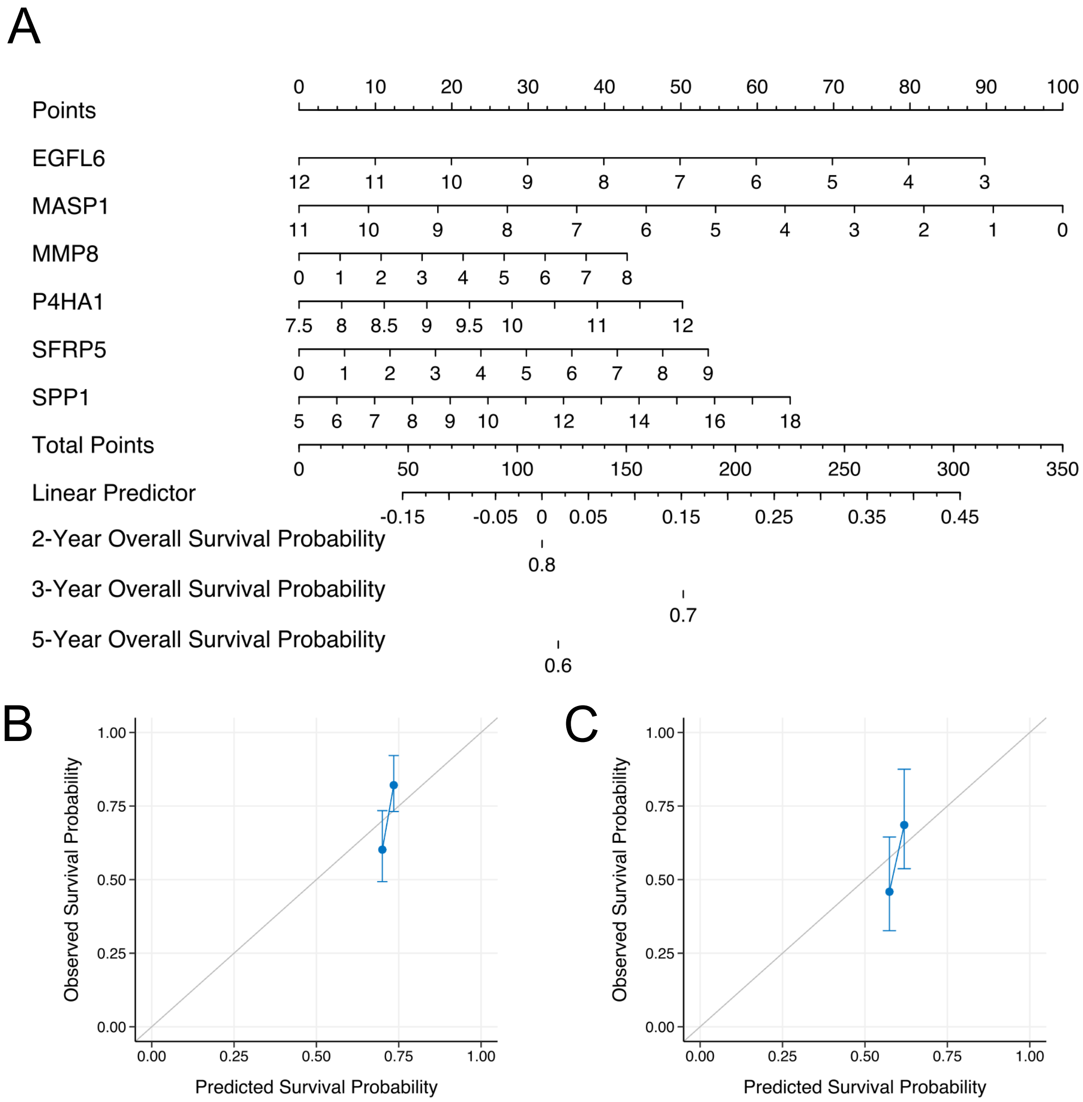

Figure S2. Nomogram and computational calibration of the model. (A) Nomogram to predict survival probability at 2, 3 and 5-year after surgery for stage HPV-, N0, patients based on the expression levels of the SCCHN TMI genes derived from the TCGA dataset. (B) Calibration curve for the nomogram when predicting 3-year overall survival. (C) Calibration curve for the nomogram when predicting 5-year overall survival.

A

Covariates	Beta	HR (95% CI for HR)	Wald test	P value
Risk groups	1.1	2.9 (1.6-5.2)	12	0.00051 ***
Age	0.027	1 (1-1.1)	4	0.045 *
Gender	0.29	1.3 (0.75-2.4)	0.96	0.33
Packyears	-0.0011	1 (0.99-1)	0.04	0.85
Alcohol per day	0.011	1 (0.94-1.1)	0.08	0.78
T category	-0.018	0.98 (0.74-1.3)	0.02	0.9

C

Covariates	Beta	HR (95% CI for HR)	Wald test	P value
Risk group	1.1	3.1 (0.97-9.9)	3.6	0.057
Age	0.05	1.1 (0.99-1.1)	3.2	0.076
Gender	0.75	2.1 (0.58-7.8)	1.3	0.25
Packyears	-0.011	0.99 (0.96-1)	0.74	0.39
Alcohol per day	0.035	1 (0.59-1.8)	0.01	0.91
T category	0.44	1.5 (0.89-2.7)	2.4	0.12

B

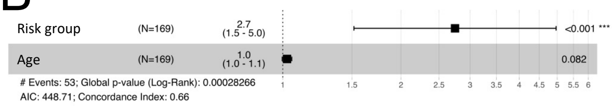

Figure S3. Univariate and multivariate analysis. The prognostic factor "risk group" was compared with other clinical confounders using univariate analysis using the Cox proportional-hazard model, as shown in the tables. (A) Outcomes of the univariate analysis of the SCCHN TMI using the data from TCGA. (B) The forest plots show the outcome of the multivariate analysis of the factors that were statistically significant at the univariate analysis. (C) Outcomes of the univariate analysis of the SCCHN TMI on the validation dataset (accession code GSE65858).

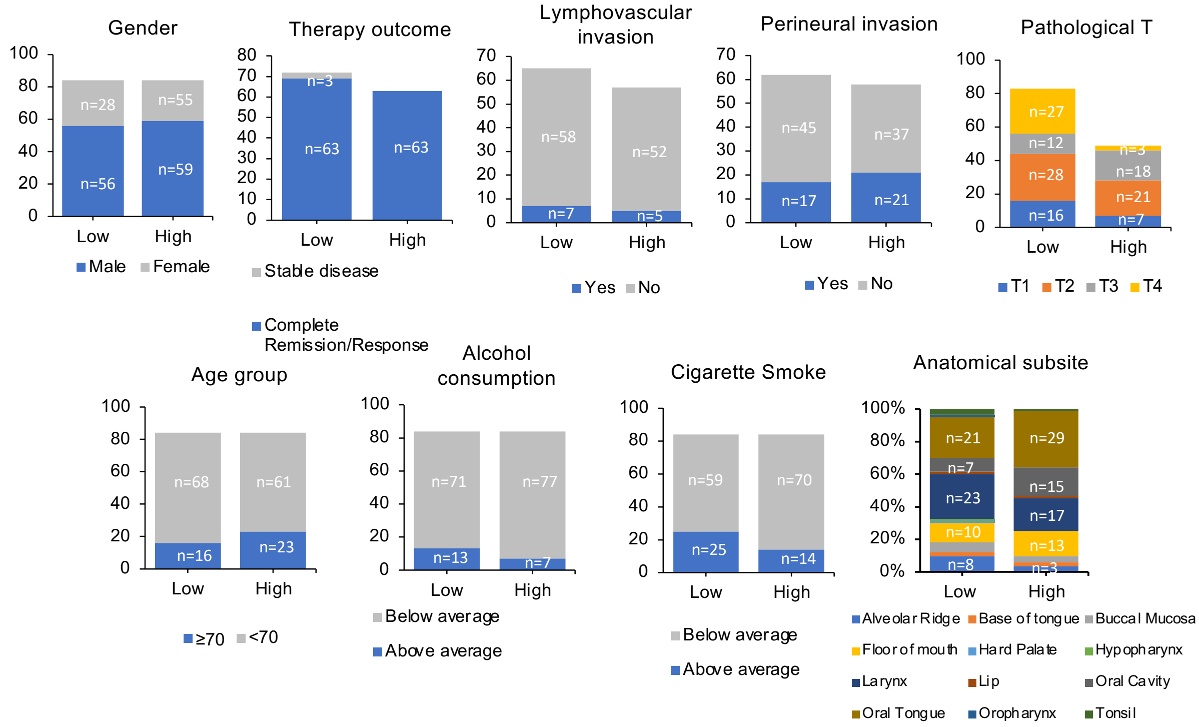

Figure S4. Comparison between low and high SCCHN TMI for the HPV^{-}, N0 sub-group among conventional clinical parameters (TCGA dataset).

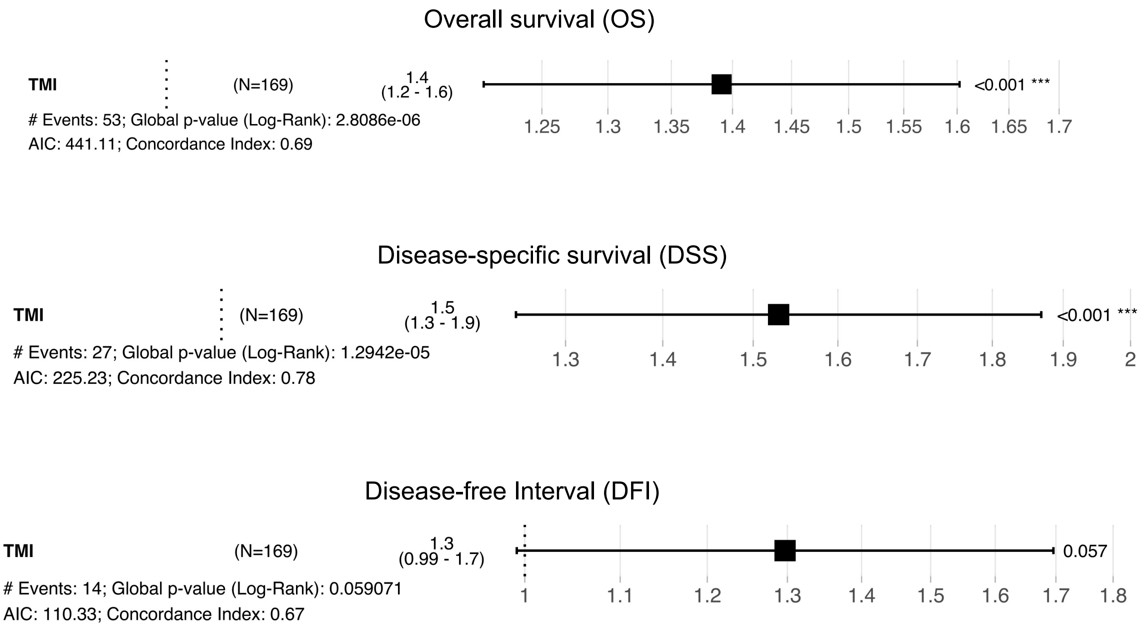

Figure S5. Association between SCCHN TMI and clinical outcomes. The hazard ratio (HR) forest plots show the effect of the SCCHN TMI on the overall survival (OS), disease-specific survival (DSS) and disease-free interval (DFI) for the HPV-, N0 cohort. Vertical dashed line corresponds with HR = 1.

A

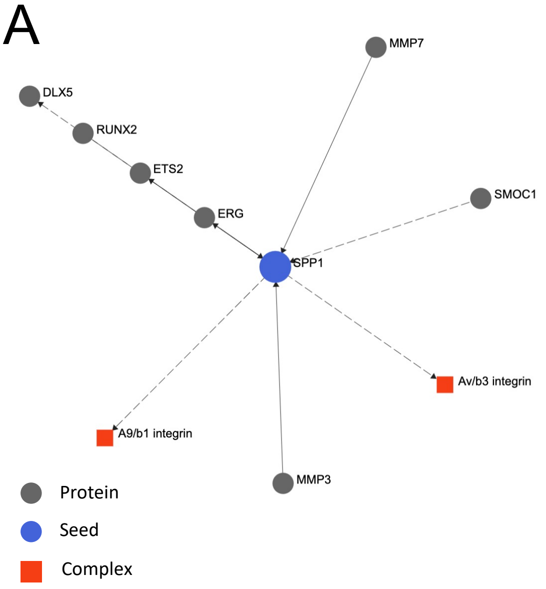

B

Pathway	Total	Expected	Hits	P.Value	FDR
Transcriptional misregulation in cancer	186	0.168	3	0.000446	0.142
Prostate cancer	97	0.0878	2	0.00314	0.499
HTLV-I infection	219	0.198	2	0.0152	1
ECM-receptor interaction	82	0.0742	1	0.0719	1
Rheumatoid arthritis	91	0.0823	1	0.0795	1
IL-17 signaling pathway	93	0.0841	1	0.0812	1
Toll-like receptor signaling pathway	104	0.0941	1	0.0904	1
TNF signaling pathway	110	0.0995	1	0.0954	1
Apelin signaling pathway	137	0.124	1	0.118	1
Signaling pathways regulating pluripotency of stem cells	139	0.126	1	0.119	1

Figure S6. Regulatory network analyses of the SCCHN TMI genes. (A) Signalling network generated via NetworkAnalyst and data from the SIGnaling Network Open Resource (SIGNOR) 2.0. The network has 10 nodes, 9 edges, and 1 seed. (B) KEGG (Kyoto Encyclopaedia of genes and Genomes) enrichment analysis.

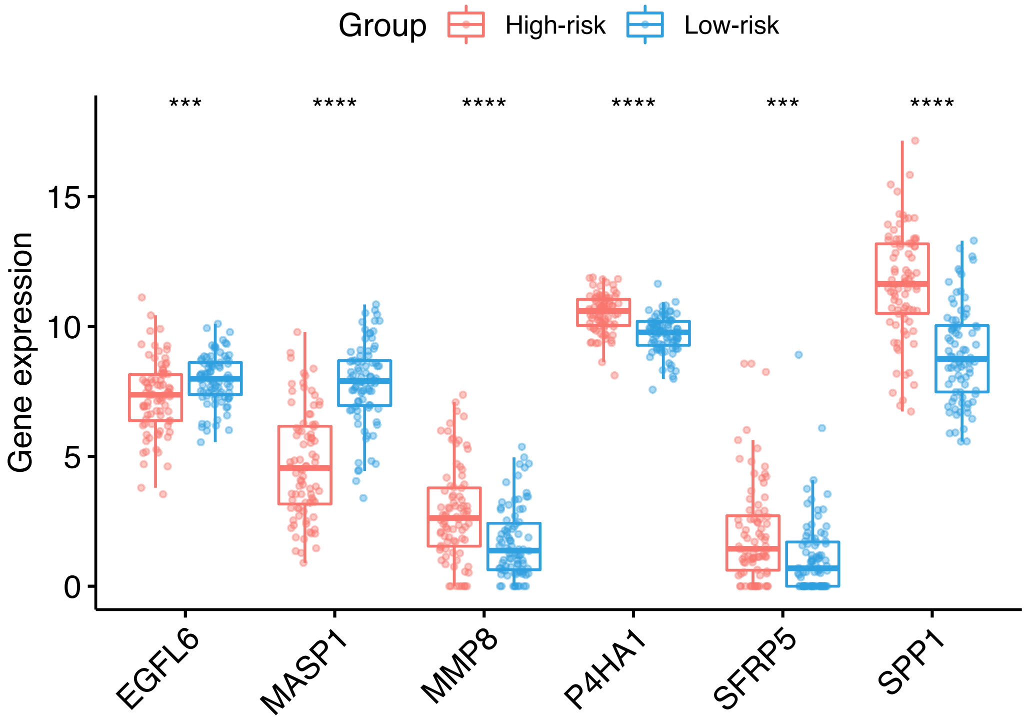

Figure S7. Expression levels as a function of the SCCHN TMI risk group for each gene. Specifically, 84 low-risk and 84 high-risk samples were compared. The size of the boxes indicates the interquartile range IQR which spans from the first quartile (Q1) to the thirst quartile (Q3). The whiskers indicate the range from Q1 + 1.5 x IQR to Q3 – 1.5 x IQR and the line is the median. Two-sided, unpaired two-samples Wilcoxon test was performed between the two groups. **** p ≤ 0.0001, *** p ≤ 0.001.
